# Maternal obesity-induced decreases in plasma, hepatic and uterine polyunsaturated fatty acids during labour is reversed through improved nutrition at conception

**DOI:** 10.1038/s41598-018-21809-9

**Published:** 2018-02-21

**Authors:** Ronan Muir, Ge Liu, Raheela Khan, Anatoly Shmygol, Siobhan Quenby, Robert Alan Gibson, Beverly Muhlhausler, Matthew Elmes

**Affiliations:** 1 Division of Nutritional Science, School of Bioscience, University of Nottingham, Sutton Bonington Campus, Loughborough, LE12 5RD England United Kingdom; 2 Graduate School of Medicine, University of Nottingham, Royal Derby Hospital, Uttoxeter Road, Derby, DE22 3DT England United Kingdom; 3 Department of Physiology, College of Medicine and Health Sciences, United Arab Emirates University, Al Ain, P. O. Box 17666 UAE; 4 Biomedical Research Unit in Reproductive Health, University Hospitals Coventry and Warwickshire NHS Trust, Coventry, CV2 2DX Warwickshire United Kingdom; 5 Healthy Mothers, Babies and Children’s Theme, South Australian Health and Medical Research Institute (SAHMRI), Adelaide, Australia; 6 Department of Wine and Food Science, FOODplus Research Centre, School of Agriculture, Food and Wine, University of Adelaide, Adelaide, Australia

## Abstract

Maternal obesity is associated with prolonged and dysfunctional labour, potentially through decreased synthesis of prostaglandins that stimulate myometrial contractions. We assessed the impact of maternal obesity on concentrations of precursor fatty acids (FA) for prostaglandin synthesis and whether any changes could be reversed by improved nutrition post-conception. Wistar rats were fed control (CON) or High-Fat, High-cholesterol (HFHC) diets 6 weeks before mating. At conception half the dams switched diets providing 4 dietary groups: (1) CON, (2) HFHC, (3) CON-HFHC or (4) HFHC-CON. During parturition rats were euthanized and FA composition of plasma, liver and uterus determined. Visceral fat was doubled in rats exposed to the HFHC diet prior to and/or during pregnancy compared to CON. HFHC diet increased MUFAs but decreased omega-3 and omega-6 PUFAs in plasma and liver. Uterine omega-3 FA concentrations were halved in HFHC versus CON rats, but all other FAs were similar. Switching from HFHC to CON diet at conception restored all FA profiles to those seen in CON rats. The increased MUFA and decreased PUFA concentrations in obese HFHC dams may contribute to aberrant prostaglandin synthesis and dysfunctional myometrial activity and it may be possible to reverse these changes, and potentially improve labour outcomes, by improving nutrition at conception.

## Introduction

Between the years 1975–2014 global obesity levels have increased from 105 to 641 million people^1^. The UK level of obesity in women of reproductive age currently stands at 20% and is estimated to reach 50% by 2050^2^ and this is associated with a parallel increase in the number of women entering pregnancy with a high BMI (Centre for Maternal and Child Enquiries (3). Maternal obesity increases the risk of gestational diabetes, pre-eclampsia, post-partum haemorrhage and prolonged and dysfunctional labour resulting in emergency caesarean delivery^3–6^. Although the increased risks of adverse pregnancy outcomes in obese women is well established, very few studies have attempted to elucidate the underlying mechanism(s), and therefore identify potential strategies for intervention.

Prostaglandins PGF_2α_, PGE_2_ and PGI_2_ produced within intrauterine tissues play a central role in regulating uterine activity during pregnancy and parturition^7^, and increased production of these compounds is required for the initiation and progression of labour. PGE_2_ maintains uterine quiescence during pregnancy but stimulates myometrial contractions during labour via specific EP receptors^8^ whereas PGF_2α_ stimulates myometrial contractions^9^ and PGI_2_ primes the myometrium for enhanced contractile response^10^. These 2 series prostaglandins are, in turn, derived from the omega-6 fatty acid arachidonic acid (AA 20:4 n-6). In contrast, the omega-3 fatty acid, eicosapentaenoic acid (EPA 20:5 n-3) gives rise to the 3 series prostaglandins, which are less biologically active and thus reduce uterine contractility and delay parturition. AA is either acquired from the diet or synthesized *de novo* from the precursor n-6 PUFA linoleic acid (LA, 18:2 n-6)^11,12^.

The fact that the omega-3 and omega-6 fats give rise to PGs with distinct biological activities has led to suggestions that the dietary balance of omega-6 and omega-3 fatty acids may play a key regulatory role in labour and parturition, particularly the physiological processes requiring prostaglandin synthesis. This is supported by human and animal studies that have shown a significant increase in the risk of premature labour when humans or animal consume diets rich in n-6 PUFA^13,14^. In contrast, diets high in n-3 PUFA significantly prolong gestation and labour^15–20^. Dietary alteration of prostaglandin synthesis has been proposed to occur through n-3 and n-6 PUFAs, such as DGLA (20:3, n-6) and EPA competing with AA for incorporation into phospholipids of the plasma membrane^21,22^ but also processing by desaturase^23,24^ and COX-2 enzymes that synthesise long chain PUFAs and prostaglandins respectively.

The role of altered fatty acid composition as a contributing factor to the increased risk of pregnancy complications in obese women has not been previously explored. This is despite clear evidence of the importance of dietary fatty acids in regulating the physiology and endocrinology of female reproduction and the timing and length of labour^25^, and evidence of altered prostaglandin production in the context of maternal obesity. We have recently established that plasma levels of PGF_2α_ during labour are significantly lower in obese high-fat, high-cholesterol (HFHC) fed rats compared to lean controls^26^ suggesting that maternal obesity is associated with a decreased capacity for PGF_2α_ synthesis. In addition, modulation of both monounsaturated fatty acids (MUFAs) and omega-3 and omega-6 polyunsaturated fatty acids (PUFAs) have been reported to affect the timing of parturition^27–29^ and may thus play a key role in the prolonged and dysfunctional labour often associated with maternal obesity^4^.

Consequently, the aim of the present study was to investigate the effects of dietary HFHC induced maternal obesity on plasma, hepatic and uterine fatty acid composition during labour, with a particular focus on the precursor fatty acids for prostaglandin synthesis. A second aim was to ascertain whether changing diet at conception can improve the n-6 fatty acid status and increase the capacity for PGF_2α_ synthesis, and thus identify a potential intervention for reducing the risk of prolonged and dysfunctional labour associated with maternal obesity.

## Results

### Body and Fat Depot Weights

**Figure 1:**
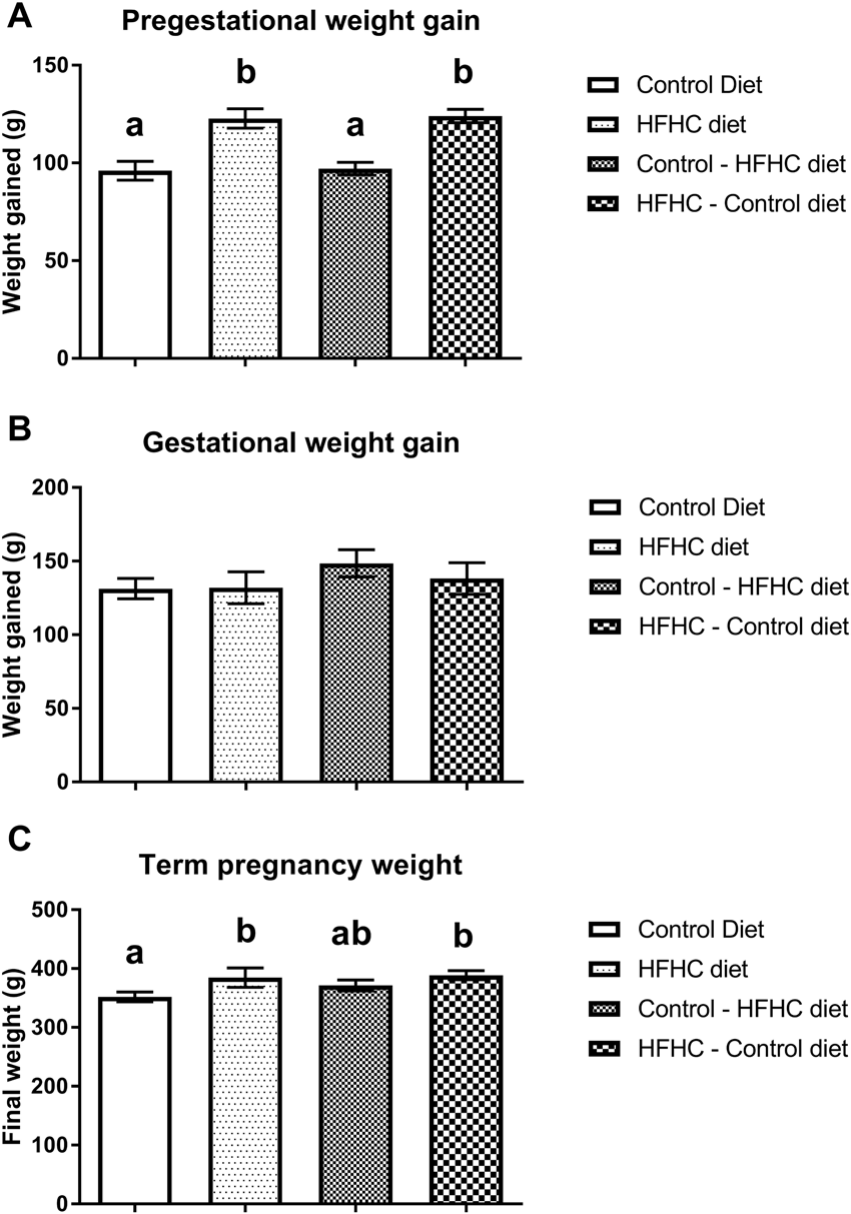
Bodyweight changes 6 weeks before mating (**A**) during pregnancy (**B**) and final weight at term pregnancy (**C**) in rats fed either a CON, HFHC, CON-HFHC or HFHC-CON diet. Values are means with SEM represented by vertical bars. Dietary treatment groups with different superscript letters are significantly different at the P < 0.05 level.

**Figure 2:**
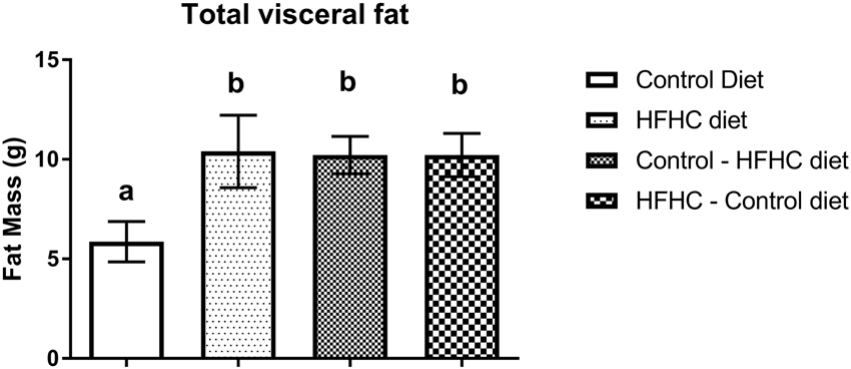
The effects of feeding a CON, HFHC, CON-HFHC or HFHC-CON diet on maternal total visceral fat depot weights at term pregnancy. Values are means with SEM represented by vertical bars. Dietary treatment groups with different superscript letters are significantly different at the P < 0.05 level.

Feeding a HFHC diet for 6 weeks prior to mating was associated with a significant increase in body weight at conception (Fig. 1A). There was no difference in gestational weight gain between the 4 treatment groups (Fig. 1B). At the time of parturition, however, rats fed the HFHC diet before mating were still significantly heavier than rats fed the CON diet before mating, independent of their diet post-conception (P < 0.05). Body weight of the rats fed the CON diet before mating, but switched to the HFHC diet at conception (CON-HFHC) at the end of gestation was not different to those in any other treatment group (Fig. 1C). Visceral fat mass (sum of perirenal and gonadal fat) was 2-fold higher in rats exposed to the HFHC diet either before and/or post-conception compared to the CON group (Fig. 2).

### Fatty acid composition of the plasma

The fatty acid profile of the plasma collected from rats fed a CON or HFHC diet for 6 weeks prior to and during pregnancy and those whose diet was switched at conception is presented in Table 1.

**Table 1:** A comparison of the fatty acid composition of maternal plasma from rats fed either a control chow (CONTROL), High-Fat, High-Cholesterol (HFHC), CON-HFHC, or HFHC-CON diet. Control and HFHC diets were fed 6 weeks prior to and during the whole of pregnancy. CON-HFHC and HFHC-CON exposed rats were fed CONTROL and HFHC diets prior to pregnancy but then switched to HFHC and CON diets at conception respectively and maintained on the switched diet until parturition.

Plasma	CONTROL (n = 5)	HFHC (n = 6)	CONTROL-HFHC (n = 6)	HFHC-CONTROL (n = 5)	P value
	Mean ± SEM	Mean ± SEM	Mean ± SEM	Mean ± SEM	
**Total Saturates**	34.45 ± 0.53^**A**^	33.83 ± 0.40^**A**^	33.32 ± 0.75^**A**^	36.04 ± 0.26^B^	**0.009**
14:0	0.36 ± 0.01^**A**^	1.39 ± 0.12^**B**^	1.36 ± 0.16^**B**^	0.53 ± 0.04^**A**^	**<0.0001**
15:0	0.20 ± 0.01^**A**^	0.38 ± 0.02^**B**^	0.37 ± 0.02^**B**^	0.27 ± 0.01^**C**^	**<0.0001**
16:0	20.40 ± 0.75	21.01 ± 0.25	20.10 ± 0.67	20.70 ± 0.23	0.592
17:0	0.44 ± 0.03	0.54 ± 0.05	0.48 ± 0.05	0.46 ± 0.01	0.260
18:0	11.85 ± 0.95^**A**^	9.67 ± 0.30^**B**^	10.22 ± 0.15^**B**^	12.71 ± 0.25^**A**^	**0.001**
20:0	0.14 ± 0.02	0.13 ± 0.02	0.13 ± 0.01	0.16 ± 0.02	0.919
22:0	0.24 ± 0.03^**A**^	0.13 ± 0.02^**B**^	0.13 ± 0.01^**B**^	0.25 ± 0.01^**A**^	**0.010**
24:0	0.22 ± 0.02	0.14 ± 0.02	0.15 ± 0.01	0.22 ± 0.01	0.166
**Total Monos**	9.39 ± 0.96^**A**^	27.10 ± 1.33^**B**^	24.28 ± 2.30^**B**^	9.58 ± 0.31^**A**^	**<0.0001**
**Total Omega 9**	7.57 ± 0.91^**A**^	23.53 ± 0.97^**B**^	21.37 ± 2.04^**B**^	7.75 ± 0.26^**A**^	**<0.0001**
18:1n-9	7.51 ± 0.90^**A**^	23.37 ± 0.96^**B**^	21.24 ± 2.04^**B**^	7.75 ± 0.26^**A**^	**<0.0001**
20:1n-9	0.10 ± 0.02^**A**^	0.16 ± 0.02^**B**^	0.13 ± 0.01^**B**^	—	**<0.0001**
**Total Omega 7**	1.81 ± 0.08^**A**^	3.57 ± 0.41^**B**^	2.91 ± 0.28^**B**^	1.83 ± 0.07^**A**^	**<0.0001**
16:1n-7	0.70 ± 0.06^**A**^	2.36 ± 0.37^**B**^	1.86 ± 0.22^**B**^	0.84 ± 0.09^**A**^	**<0.0001**
18:1n-7	1.11 ± 0.04^**AB**^	1.21 ± 0.04^**A**^	1.05 ± 0.05^**BC**^	0.99 ± 0.03^**C**^	**0.005**
**Total Omega 3**	9.77 ± 0.68^**A**^	3.03 ± 0.33^**B**^	4.49 ± 0.35^**C**^	8.99 ± 0.34^**A**^	**<0.0001**
18:3n-3	1.12 ± 0.12^**A**^	0.38 ± 0.03^**B**^	0.62 ± 0.04^**C**^	0.85 ± 0.06^**D**^	**<0.0001**
20:5n-3	0.19 ± 0.02^**AB**^	0.08 ± 0.01^**C**^	0.11 ± 0.02^**BC**^	0.17 ± 0.03^**A**^	**0.007**
22:5n-3	1.05 ± 0.12^**A**^	0.31 ± 0.07^**B**^	0.44 ± 0.11^**B**^	0.77 ± 0.02^**C**^	**<0.0001**
22:6n-3	7.42 ± 0.62^**A**^	2.25 ± 0.28^**B**^	3.34 ± 0.30^**B**^	7.20 ± 0.33^**A**^	**<0.0001**
**Total Omega 6**	46.35 ± 0.80^**A**^	35.20 ± 1.17^**B**^	36.92 ± 1.69^**B**^	45.23 ± 0.38^**A**^	**<0.0001**
18:2n-6	22.02 ± 1.48^**A**^	17.28 ± 0.31^**B**^	19.02 ± 0.61^**B**^	19.17 ± 0.66^**B**^	**0.008**
18:3n-6	0.59 ± 0.05	0.49 ± 0.05	0.53 ± 0.05	0.65 ± 0.04	0.210
20:2n-6	0.26 ± 0.01^**AC**^	0.20 ± 0.02^**B**^	0.20 ± 0.01^**B**^	0.23 ± 0.03^**BC**^	**0.045**
20:3n-6	0.44 ± 0.05	0.48 ± 0.02	0.41 ± 0.03	0.44 ± 0.03	0.412
20:4n-6	18.39 ± 1.2^**A**^	11.57 ± 0.81^**B**^	12.71 ± 1.01^**B**^	19.63 ± 0.70^**A**^	**<0.0001**

#### Saturated and monounsaturated fatty acids

Total plasma saturated fatty acid levels at partition were higher in the HFHC-CON group compared to all other dietary treatments (P < 0.009), but were not different between the CON, HFHC and CON-HFHC groups. Analysis of the individual saturated fatty acids identified that exposure to a HFHC either during the entire period, or only post-conception, was associated with significant increases in levels of myristic acid (14:0) and pentadecylic acid (15:0) in comparison to the CON group. These increases were partially (in the case of pentadecylic acid) or completely (in the case of myristic acid) normalised by switching rats fed a HFHC diet before mating to a CON diet after conception. Conversely, plasma levels of stearic (18:0) and behenic Acid (22:0) were lower in the CON-HFHC and HFHC rats when compared to CON, but were not different between the HFHC-CON and CON treatment groups.

Total plasma monounsaturated fatty acid concentrations, as well as concentrations the major omega-9 and -7 monounsaturated fatty acids, oleic acid (18:1 n-9) and palmitoleic acid (16:1 n-7), were increased by 3-fold in rats exposed to HFHC vs CON diet during the post-conception period, independent of their diet prior to mating (Table 1). Plasma concentrations of vaccenic acid (18:1 n-7), however, were not different between CON, a HFHC, and CON-HFHC dietary groups, but were lower in the CON-HFHC and HFHC-CON fed rats compared to those exposed to the HFHC diet during both the pre-conception and post-conception periods.

#### Omega-3 and omega-6 polyunsaturated fatty acids

Plasma total omega-3 PUFA levels at parturition were lower in rats fed the HFHC diet post-conception, independent of their diet before mating (Table 1). The same pattern was also observed for the individual omega-3 LCPUFA, EPA and DHA. Thus, feeding rats the HFHC diet during gestation was associated with reduced plasma EPA and DHA contents, however switching rats from a HFHC diet to a CON diet post-conception ameliorated these effects (Table 1). In the case of ALA, there appeared to be a dose-response relationship between HFHC exposure and plasma concentrations, such that plasma levels were lowest in rats fed the HFHC diet both before and after conception, highest in rats fed the CON diet during both periods and intermediate in the other two treatment groups (Table 1).

Total Omega-6 PUFA concentrations and concentrations of the omega-6 LCPUFA, AA, followed a similar pattern, and were lower in rats exposed to the HFHC diet compared to the CON diet post-conception (All P ≤ 0.007). Plasma LA (18:2 n-6), concentrations were lower in rats fed the HFHC diet either pre- or post-conception compared to the CON group, and were not restored to CON levels by switching HFHC rats to the CON diet after mating.

### Fatty acid composition of the liver

#### Saturated and monounsaturated fatty acids

The fatty acid composition of the liver followed a similar pattern to that of the plasma (Table 2). Total saturated fat content was lower in rats exposed to the HFHC diet post-conception, independent of their diet prior to mating. Exposure to the HFHC diet post-conception increased liver myristic (14:0) and pentadecyclic (15:0) acid contents ≥ 2-fold when compared to CON, and switching from a HFHC to control diet post-conception completely reversed this effect in HFHC-CON animals. Liver palmitic acid (C16:0) content was lower in rats in the CON-HFHC vs CON treatment groups, but was not different between the CON, HFHC and HFHC-CON groups. Stearic (18:0), behenic (22:0) and lignoceric (24:0) acid were also lower in rats fed a HFHC diet post-conception, but were not different between the HFHC-CON and CON groups.

**Table 2:** A comparison of the fatty acid composition of maternal liver from rats fed either a control chow (CONTROL), High-Fat, High-Cholesterol (HFHC), CON-HFHC, or HFHC-CON diet. Control and HFHC diets were fed 6 weeks prior to and during the whole of pregnancy. CON-HFHC and HFHC-CON exposed rats were fed CONTROL and HFHC diets prior to pregnancy but then switched to HFHC and CON diets at conception respectively and maintained on the switched diet until parturition.

Liver	CONTROL (n = 5)	HFHC (n = 6)	CONTROL-HFHC (n = 6)	HFHC-CONTROL (n = 5)	P value
	Mean ± SEM	Mean ± SEM	Mean ± SEM	Mean ± SEM	
Total Saturates	43.45 ± 0.77^A^	34.48 ± 0.73^B^	35.63 ± 0.90^B^	42.94 ± 0.63^A^	**<0.0001**
14:0	0.43 ± 0.02^A^	1.32 ± 0.31^B^	0.97 ± 0.08^B^	0.52 ± 0.12^A^	0.016
15:0	0.19 ± 0.02^A^	0.33 ± 0.03^B^	0.30 ± 0.02^B^	0.18 ± 0.02^A^	0.002
16:0	22.48 ± 0.83^A^	21.96 ± 0.24^AB^	20.86 ± 0.35^B^	21.73 ± 0.62^AB^	0.007
17:0	0.45 ± 0.03	0.45 ± 0.03	0.43 ± 0.02	0.43 ± 0.02	0.833
18:0	17.91 ± 0.77^A^	9.61 ± 0.45^B^	12.17 ± 0.63^C^	18.61 ± 0.62^A^	**<0.0001**
20:0	0.20 ± 0.04	0.13 ± 0.03	0.11 ± 0.02	0.19 ± 0.08	0.615
22:0	0.40 ± 0.04^A^	0.14 ± 0.03^B^	0.17 ± 0.03^BC^	0.29 ± 0.09^AC^	0.026
24:0	0.38 ± 0.03^A^	0.16 ± 0.02^B^	0.20 ± 0.03^B^	0.32 ± 0.04^A^	**<0.0001**
Total Monos	6.94 ± 0.32^A^	31.02 ± 1.87^B^	23.89 ± 1.60^C^	7.03 ± 0.49^A^	**<0.0001**
Total Omega 9	4.89 ± 0.24^A^	27.29 ± 1.62^B^	21.19 ± 1.54^C^	5.27 ± 0.43^A^	**<0.0001**
18:1n-9	4.58 ± 0.31^A^	27.01 ± 1.63^B^	20.98 ± 1.52^C^	5.06 ± 0.41^A^	**<0.0001**
20:1n-9	—	0.21 ± 0.01	0.15 ± 0.01	0.10 ± 0.03	0.072
22:1n-9	—	0.07 ± 0.02	0.08 ± 0.02	0.13 ± 0.06	0.344
Total Omega 7	1.92 ± 0.08^A^	3.67 ± 0.32^B^	2.66 ± 0.08^C^	1.66 ± 0.04^A^	**<0.0001**
16:1n-7	0.65 ± 0.08^A^	2.15 ± 0.28^B^	1.43 ± 0.07^C^	0.48 ± 0.03^A^	**<0.0001**
18:1n-7	1.27 ± 0.06^A^	1.52 ± 0.07^B^	1.24 ± 0.03^A^	1.18 ± 0.04^A^	0.010
Total Omega 3	13.99 ± 0.46^A^	3.30 ± 0.44^B^	5.91 ± 0.34^C^	13.02 ± 0.49^A^	**<0.0001**
18:3n-3	0.49 ± 0.06	0.31 ± 0.03	0.43 ± 0.06	0.45 ± 0.05	0.100
20:5n-3	0.17 ± 0.02^A^	0.07 ± 0.02^B^	0.10 ± 0.01^BC^	0.15 ± 0.03^AC^	0.014
22:5n-3	1.14 ± 0.08^A^	0.30 ± 0.07^B^	0.49 ± 0.08^C^	0.92 ± 0.02^A^	**<0.0001**
22:6n-3	12.19 ± 0.45^A^	2.62 ± 0.37^B^	4.89 ± 0.44^C^	11.50 ± 0.50^A^	**<0.0001**
Total Omega 6	35.60 ± 0.58^AC^	30.39 ± 1.16^B^	33.64 ± 0.69^A^	36.75 ± 0.61^C^	**<0.0001**
18:2n-6	13.08 ± 0.56^A^	15.65 ± 0.40^B^	16.14 ± 1.20^B^	11.86 ± 0.39^A^	0.001
18:3n-6	0.56 ± 0.13	0.46 ± 0.07	0.49 ± 0.07	0.61 ± 0.16	0.858
20:2n-6	0.45 ± 0.05^A^	0.24 ± 0.01^B^	0.27 ± 0.02^B^	0.43 ± 0.06^A^	0.008
20:3n-6	0.52 ± 0.05	0.40 ± 0.04	0.39 ± 0.04	0.38 ± 0.08	0.241
20:4n-6	14.32 ± 0.64^A^	7.89 ± 0.48^B^	10.77 ± 0.59^C^	15.88 ± 0.62^A^	**<0.0001**

The most substantial shifts in hepatic fatty acid composition were observed in the monounsaturated fatty acid fraction, with total hepatic monounsaturated fatty acid content increased more than 4-fold in rats fed the HFHC diet post-conception, independent of their diet before mating (Table 2). Hepatic monounsaturated fatty acid content in rats switched from a HFHC diet to a control diet at conception was, however, not different to the CON group. Hepatic omega-9 and omega-7 monounsaturated fatty acid contents within the liver followed a similar pattern to total monounsaturated fatty acid contents. The individual omega-9 and omega-7 monounsaturated fatty acids significantly altered by diet in this way were oleic (18:1 n-9) and palmitoleic acids (16:1 n-7). Interestingly, the omega 7 fatty acid vaccenic acid (18:1, n-7) did not follow the same trend, and concentrations of this fatty acid were higher in HFHC rats in comparison to the CON, CON-HFHC, and HFHC-CON dietary groups.

#### Omega-3 and omega-6 polyunsaturated fatty acids

Hepatic total omega-3 content was 4-fold lower in rats fed the HFHC diet post-conception, compared to both the CON and HFHC-CON groups (Table 2). Rats switched from the control to HFHC diet at conception (CON-HFHC) had a hepatic omega-3 content that was intermediate between the HFHC-CON, CON and HFHC groups (Table 2). There was no effect of the HFHC diet on hepatic ALA concentrations, but concentrations of EPA, DPA and DHA were all lower in rats exposed to the HFHC diet after conception compared to the CON treatment group. In particular, DHA levels were reduced approximately 5-fold in HFHC fed rats compared to the CON group. Rats that were switched from a CON to HFHC diet at conception had lower EPA, DHA and DPA contents than CON rats, but DPA and DHA levels were still significantly higher than in rats fed the HFHC diets both before and after conception (HFHC). Conversely, EPA, DPA and DHA concentrations in rats switched from a HFHC diet to a CON diet at conception were similar to those in CON animals.

Total hepatic omega 6 polyunsaturated fatty acid levels of the liver were approximately 5% lower in HFHC rats compared to the CON group, but was not different between the CON, CON-HFHC, and HFHC-CON groups. In terms of individual omega-6 fatty acids, gamma linoleic (18:3 n-6) and di-homo-gamma-linoleic (20:3 n-6) acid were not significantly different among the 4 dietary groups, while other major omega-6 PUFA were significantly altered. Thus, hepatic LA content was increased in rats exposed to a HFHC diet post-conception when compared to CON rats, but was not different between the HFHC-CON and CON groups. In contrast, exposure to the HFHC diet during pregnancy significantly decreased hepatic levels of both eicosadienoic (20:2, n-6) and AA when compared to the CON group, but were not different between the HFHC-CON and CON groups. Interestingly there also appeared to be a dose-response relationship between the duration of HFHC exposure and hepatic AA concentrations, such that levels were lowest in rats fed the HFHC diet both before and after conception, highest in rats fed the CON diet during both periods and intermediate in the CON-HFHC and HFHC-CON groups.

### Fatty acid composition of the uterus

Uterine composition of fatty acids are presented in Table 3. The uterus was particularly resistant to any change in fatty acid composition, and there were no differences in the content of saturated, monounsaturated or omega-6 polyunsaturated fatty acids between the 4 treatment groups. Total omega-3 fatty acid content in the uterine horn was, however, ~2-fold lower in the HFHC group compared to the CON group (P < 0.022). Importantly, rats switched from a HFHC diet to a control diet at conception had omega-3 fatty acid concentrations in their uterine horn that were not different from the CON group. The shifts in uterine omega-3 concentrations appeared to be driven primarily by changes in DHA, which tended (P = 0.095) to follow a similar pattern to that for total omega-3 concentration (Table 3).

**Table 3:** A comparison of the fatty acid composition of uterine horn from rats fed either a control chow (CONTROL), High-Fat, High-Cholesterol (HFHC), CON-HFHC, or HFHC-CON diet. Control and HFHC diets were fed 6 weeks prior to and during the whole of pregnancy. CON-HFHC and HFHC-CON exposed rats were fed CONTROL and HFHC diets prior to pregnancy but then switched to HFHC and CON diets at conception respectively and maintained on the switched diet until parturition.

Uterus	CONTROL (n = 5)	HFHC (n = 6)	CONTROL-HFHC (n = 6)	HFHC-CONTROL (n = 5)	P value
	Mean ± SEM	Mean ± SEM	Mean ± SEM	Mean ± SEM	
**Total Saturates**	40.6 ± 5.2	46.1 ± 1.7	45.2 ± 4.6	44.7 ± 3.5	0.6530
14:0	1.3 ± 0.3	2.2 ± 0.3	1.7 ± 0.2	1.6 ± 0.2	0.0980
15:0	0.3 ± 0.0	0.5 ± 0.1	0.5 ± 0.1	0.4 ± 0.0	0.2090
16:0	24.3 ± 1.2	23.9 ± 1.2	23.0 ± 0.8	24.4 ± 0.9	0.9090
17:0	1.1 ± 0.3	1.0 ± 0.2	1.2 ± 0.3	1.0 ± 0.2	0.6710
18:0	5.4 ± 3.1	10.8 ± 2.8	10.5 ± 3.1	9.4 ± 3.7	0.6470
20:0	0.8 ± 0.3	0.7 ± 0.1	0.9 ± 0.2	0.8 ± 0.1	0.5560
22:0	1.6 ± 0.5	1.4 ± 0.3	1.5 ± 0.4	1.4 ± 0.3	0.9000
24:0	1.3 ± 0.4	1.2 ± 0.3	1.3 ± 0.4	1.3 ± 0.3	0.8860
**Total Monos**	15.2 ± 2.8	17.2 ± 2.4	15.1 ± 1.9	15.3 ± 3.4	0.8540
**Total Omega 9**	12.5 ± 2.4	14.3 ± 2.1	13.0 ± 1.4	12.2 ± 2.9	0.8800
18:1n-9	12.4 ± 2.3	14.2 ± 2.0	12.8 ± 1.3	12.1 ± 2.8	0.2110
20:1n-9	0.3	0.3 ± 0.0	0.2 ± 0.0	0.2 ± 0.0	0.6550
22:1n-9	—	0.1 ± 0.2	0.2 ± 0.0	—	0.5340
**Total Omega 7**	2.7 ± 0.5	2.8 ± 0.4	2.1 ± 0.6	3.1 ± 0.5	0.3100
16:1n-7	1.1 ± 0.2	1.6 ±0.3	1.2 ± 0.3	1.5 ± 0.4	0.4210
18:1n-7	1.8 ± 0.2	1.5 ± 0.1	1.6 ± 0.2	1.8 ± 0.1	0.1330
**Total Omega 3**	4.8 ± 0.7^A^	2.8 ± 0.3^B^	4.1 ± 0.2^AB^	4.2 ± 0.5^A^	**0.0220**
18:3n-3	0.9 ± 0.4	0.6	1.1 ± 0.4	0.6 ± 0.1	0.1990
20:5n-3	0.2 ± 0.1	0.2 ± 0.0	0.2 ± 0.0	0.4 ± 0.2	0.5140
22:5n-3	0.9 ± 0.1	0.8 ± 0.1	1.0 ± 0.1	0.8 ± 0.1	0.3040
22:6n-3	3.1 ± 0.7	1.8 ± 0.2	2.3 ± 0.1	3.2 ± 0.5	0.0950
**Total Omega 6**	39.2 ± 2.8	33.0 ± 1.3	34.7 ± 2.6	35.4 ± 2.2	0.2450
18:2n-6	15.0 ± 4.8	9.1 ± 1.3	13.1 ± 3.2	10.5 ± 2.0	0.4680
18:3n-6	2.2 ± 0.7	1.7 ± 0.4	1.5 ± 0.2	1.5 ± 0.3	0.7580
20:2n-6	0.9 ± 0.1	0.6 ± 0.1	0.6 ± 0.0	0.8 ± 0.1	0.1600
20:3n-6	2.4 ± 0.6	2.0 ± 0.4	2.2 ± 0.7	1.6 ± 0.2	0.6490
20:4n-6	12.5 ± 3.0	12.2 ± 1.2	11.2 ± 1.1	13.7 ± 1.9	0.9210

## Discussion

The overall aim of the current study was to investigate the effects of HFHC feeding to induce obesity and its subsequent effects on the fatty acid composition of the plasma, liver and uterus. As expected, feeding the HFHC diet significantly increased the weight of the dams both pre-gestation and during pregnancy. The increases in bodyweight was attributed to significant increases in the accumulation of visceral fat, which was twice as high in rats exposed to the HFHC diet either before or after conception compared to rats fed a control diet during this entire period.

With confirmation that the HFHC diet causes maternal obesity, we wanted to determine its impact on plasma, hepatic and uterine fatty acid composition and whether it can be reversed by a dietary change at conception. What is evident from the current study is that exposure to the HFHC diet significantly alters the fatty acid profile of the plasma, liver and, to a more limited extent, the uterus. The fatty acid profile of both the plasma and liver from HFHC fed rats was characterised by significant increases in MUFAs with parallel decreases in total omega-3 and omega-6 PUFAs when compared to CON fed rats, findings consistent with previous studies utilising maternal high fat diets in rats and non-human primates^30,31^ as well as the fatty acid status of obese adults^32,33^ and obese pregnant women at mid-gestation^34^. Critically, we have established that switching diets at conception from a CON or HFHC diet and feeding a HFHC or CON diet during pregnancy respectively, reversed the plasma fatty acid profiles, such that these mirrored those of the post-conception diets. This suggests that alterations in circulating and hepatic fatty acid composition in rats consuming an obesogenic HFHC diet before conception, can be favourably altered by switching to a CON diet during pregnancy.

One intriguing finding was that exposure to a HFHC diet significantly altered the fatty acid profiles of the plasma and liver, but induced very little change in the uterus. The uterus only differed in total omega-3 fatty acids, which was reduced by half by the HFHC diet, and this effect was reversed when rats were switched to a CON diet post-conception. It is plausible that the uterus could be resistant to changes in dietary fatty acids, similar to the brain^35^. A further possibility could be that by collecting tissue from labouring animals the fatty acids within the uterus necessary for labour had been utilised, and differences may only be apparent prior to the onset of labour. Nevertheless, a decrease in omega-3 fatty acids in the uterus following feeding of the HFHC diet has the potential to compromise uterine CAP expression by altering the fluidity of the plasma membrane^4^. Fatty acids play a vital role in the formation of the plasma membrane lipid bilayer, and increases in membrane PUFA content is associated with increased membrane fluidity, which in turn affects the expression, function, and downstream signalling of integral proteins^35^. This theory is supported by the findings of de Jonge *et al*.^36^ where the membrane fluidity of cultured rat ventricular myocytes decreases following incorporation of the omega-3 fatty acid EPA and that this is associated with increases in the regularity of their spontaneous contractions. It was also evident in the same study that ventricular myocytes treated with endothelin 1 to stimulate a phospholipase-C β response (PLC- β) was significantly increased following EPA treatment when compared to myocytes grown in control medium. These research findings are significant in respect to the current study because endothelin-1 causes potent constriction of myometrial strips from term human pregnancies^37^ and the PLC signalling pathway plays an important role in regulating uterine contractile activity. As a result, a plausible deduction would be that the halving of the total omega-3 fatty acids within the uterus following exposure to the HFHC diet could be playing a key role in the un-coordinated *ex vivo* contractions previously observed in our maternal obesity model^38^.

An additional mechanism through which fatty acids can impact on the physiology of female reproduction is through alterations in prostaglandin synthesis, particularly synthesis of the 2 series prostaglandins PGF_2α_ and PGE_2_ which can both stimulate^8,39^ and relax^8^ myometrial contractions respectively. The omega-6 PUFA AA is the direct precursor for synthesis of the 2 series prostaglandins and there is vast evidence to show that dietary manipulation of omega-6 PUFAs can impact on PGF_2α_ and PGE_2_ production during pregnancy. Pregnant sheep fed a diet high in the essential omega-6 PUFA linoleic acid (LA) significantly increased the level of AA within plasma and placental tissues which was associated with a significant increases in both PGF_2α_ and PGE_2_ synthesis^29^ and an increased risk of premature labour^13^. In contrast, dietary supplementation with omega-3 PUFAs the fatty acid precursors for synthesis of the less potent 3-series prostaglandins PGF_3α_ and PGE_3_ reduces premature birth rates^40^. Furthermore, continual intravenous infusion of fish oil concentrate to pregnant ewes has been shown to delay and occasionally prevent glucocorticoid-induced preterm delivery^15^. Not surprisingly there has long been an interest in the use of n-3 PUFA as an effective tocolytic agent in human pregnancy. Using our model of maternal obesity we have provided evidence that a HFHC diet causes a significant reduction in plasma levels of PGF_2α_ during labour^26^ and poorly coordinated myometrial contractions *ex vivo*^38^. This significant decrease in plasma PGF_2α_ is a result of HFHC animals consuming a diet 33% lower in the availability of LA, which translates to the lower circulating levels of the prostaglandin precursor AA identified in this report (CON 18.39% ± 1.20 vs HFHC 11.57% ± 0.81; P < 0.0001). Essentially, the current study provides evidence that changing diet at conception from a HFHC to CON diet can reverse these effects and increase the plasma level of AA (HFHC-CON 19.63% ± 0.70) thereby increasing the capacity for prostaglandin synthesis and potentially improvement of labour outcomes with maternal obesity.

One of most dramatic changes observed in the current study was that following exposure to a HFHC diet there was a significant 3- & 5-fold increase in the plasma and hepatic levels of the MUFA oleic acid (18:1 n-9) respectively. Very little research has investigated the specific effects of oleic acid on prostaglandin production. However, one recently published study suggests oleic acid could also contribute to the decrease in plasma PGF_2α_ observed in our maternal obesity model^26^. Primary culture of endometrial cells from late pregnant ewes showed that oleic acid supplementation favoured PGE_2_ production and attenuated PGF_2α_ synthesis even in the presence of the prostaglandin synthesis stimulator oxytocin^27^. Interestingly, the authors speculated that raised circulating levels of oleic acid may affect the initiation and progress of parturition and could also offer a further potential mechanism behind the dysfunctional labour associated with maternal obesity. If future research can confirm inhibitory effects of oleic acid, on PGF_2α_ production then the current study shows clearly that high circulatory and hepatic levels of oleic acid caused by consumption of a HFHC diet can be decreased very easily by changing the composition of the diet consumed during pregnancy. This in turn could increase the capacity for PGF_2α_ synthesis.

It is important to consider that changes in fatty acid composition may compromise uterine contractile activity through mechanisms other than prostaglandin synthesis. One potential mechanism is the direct effect of fatty acids on uterine expression of key contraction-associated proteins (CAPs). One such protein is connexin-43 (Cx-43), which is essential for formation of gap junctions that act as intracellular conduits to facilitate cell-cell electro-coupling (synchronisation of muscular contractions) and metabolite transfer^41^. Loss of expression of Cx-43 is associated with dysfunctional myometrial contractile activity and prolonged labour^41–44^. Our previous research identified that exposure to a HFHC significantly decreases uterine expression of Cx-43 during labour^26,38^. Quantification of the phosphorylated form of Cx-43 (pCX-43, phosphorylation at serine 368) was significantly higher in labouring HFHC fed rats compared to controls^38^. This is an important observation as phosphorylation of CX-43 compromises gap junction assembly, trafficking, and intracellular communication^45^. Interestingly, cardiomyocyte contractile activity is significantly decreased when exposed to oleic acid *in vitro* by inducing gap junction disassembly as a result of phosphorylation of CX-43 by protein kinase C (PKC)^46^. Thus, the increased circulating and hepatic oleic acid levels observed in rats fed the HFHC diet in the present study could potentially contribute to the decrease in Cx-43 and increase in pCx-43 previously observed in the labouring uteri of our HFHC fed rats^38^.

To summarize, exposure to a HFHC diet to induce obesity resulted in significant shifts in plasma and hepatic fatty acid composition, but only altered levels of omega-3 fats in the uterus. HFHC feeding was associated with significant increases in MUFAs and parallel decreases in total omega-3 and omega-6 PUFAs in both the plasma and liver. In contrast the uterus was more resistant to changes in dietary fatty acid incorporation, but the uteri from HFHC exposed rats did exhibit a significant decrease in the total omega-3 fatty acids. What is very clear from the fatty acid profile of HFHC fed animals is that the diet has the capacity to detrimentally alter prostaglandin synthesis, membrane fluidity, and uterine contractile protein expression. All of the aforementioned mechanisms may play a role in prolonged and dysfunctional labour associated with maternal obesity. Of key importance is the evidence that changing from a HFHC to CON diet at conception with the absence of any reduction in maternal fat mass, reverses the fatty acid profile associated with obesity and has the potential to improve uterine contractile activity and labour outcome.

Although change of diet at conception to improve labour outcomes in obese pregnancies may be an effective approach, further research is required to determine the effects of the HFHC diet and change of diet at conception on (1) prostaglandin synthesis, (2) uterine expression of the key contractile associated proteins and (3) myometrial excitation-contraction coupling and contractility both *ex vivo and in vivo*.

## Materials and Methods

### Animals and Experimental Design

All animal experiments were performed in accordance with the relevant laws and institutional guidelines and were approved by the University of Nottingham Animal Welfare Ethical Review Board (AWERB) and the UK Home Office [Project licence code (PPL) 40/3598]. All procedures were carried out by licenced researchers under the Animals in Scientific Procedures Act (ASPA) of 1986 within the animal facilities of the University of Nottingham.

A total of 22 weanling virgin female Wistar rats (*Rattus rattus*) weighing approximately 60 g (Charles River, UK) were pair housed under normal conditions (12 hour light: dark photoperiod, 21 °C ± 5 °C room temperature, 55% ± 5% relative humidity, food and water access *ad libitum*) and randomly assigned to be fed either a standard control laboratory chow (CON, n = 11) (Harlan Laboratories, UK) or High-Fat, High-Cholesterol diet (HFHC, n = 11) as detailed in Table 4. Nutritional breakdown and fatty acid composition of the CON and HFHC diets are also provided in Tables 5 and 6. Rats were pair housed and maintained on their respective diets for 6 weeks prior to mating with stud Wistar males (Charles River, UK). Pregnancy was confirmed upon discovery of a semen plug and was recorded as gestational day 0. Once conception was confirmed half of the rat dams from each dietary group were switched to the alternate diet throughout pregnancy, such that CON fed rats were switched to a HFHC diet and HFHC fed rats to a CON chow diet respectively. This provided 4 experimental treatment groups: (1) CON (n = 5), (2) HFHC (n = 6), (3) CON-HFHC (n = 6) or (4) HFHC-CON (n = 5) diet.

**Table 4:** Detailed breakdown of ingredients used to produce 1 kg of HFHC diet. (Note that cholesterol was dissolved within in the 100 ml vegetable oil).

Constituents	Amount (g/kg)
Corn oil	100
Casein	200
Maize starch	218
Butter	295
Sucrose	100
Cellulose	50
Vitamin mix	5
Mineral mix	20
Methionine	10
Choline	2
Cholesterol	10

**Table 5:** Nutritional breakdown of the CONTROL chow and HFHC diet. Nutritional analysis was carried by Scientific Laboratory Services (SAL) Ltd. UK.

Nutritional Breakdown	CONTROL	HFHC
Energy density (Kcal/g)	3.1	5.26
Total available carbohydrates (%)	58.9	30.5
Total protein (%)	18.6	18.5
Total fat (%)	6.2	35.6
Total fibre (%)	3.5	4.8
Total ash (%)	5.3	1.9
Total moisture	7.5	8.8

**Table 6:** The fatty acid composition of the control chow and HFHC diet.

	CONTROL (%)	HFHC (%)
**Total Saturates**	20.2	46.3
14:0	0.4	7.7
15:0	0.1	0.8
16:0	14.8	26.8
17:0	0.2	0.5
18:0	3.8	9.9
20:0	0.3	0.3
22:0	0.3	0.1
24:0	0.2	0.1
**Total Monos**	20.9	29.9
16:1n-7	0.4	1.5
18:1n-9	19.0	27.6
18:1n-7	1.1	0.7
20:1n-9	0.4	0.2
22:1n-9	0.0	0.0
**Total Omega 9**	19.4	27.7
18:1n-9	19.0	27.6
20:1n-9	0.4	0.2
**Total Omega 7**	1.5	2.2
16:1n-7	0.4	1.5
18:1n-7	1.1	0.7
**Total Omega 3**	6.0	0.8
18:3n-3	6.0	0.7
**Total Omega 6**	52.8	19.8
18:2n-6	52.7	19.6
20:2n-6	0.1	0.0
20:3n-6	0.0	0.1
20:4n-6	0.0	0.1
**n-6/n-3 ratio**	8.8	24.75

All pregnant rats were then individually housed and maintained on their respective diet throughout gestation. Daily food intake and weight gain measurements were recorded prior to and during pregnancy. At gestational day 22, hourly checks were made for signs of parturition and following birth of the 5^th^ pup each rat dam was immediately euthanized by CO_2_ asphyxiation and cervical dislocation. Pups were euthanased by overdose of pentobarbitone and cervical dislocation. Maternal blood was immediately collected via cardiac puncture into EDTA coated tubes (Sarstedt, Nümbrecht, Germany), and subsequently centrifuged at 13,000 rpm and the plasma snap frozen in liquid nitrogen and stored at −80 °C. The uterus, liver, gonadal and perineal fat depots were dissected out, weighed, snap frozen in liquid nitrogen and stored at −80 °C for future analysis.

### Lipid Extraction

Prior to lipid extraction, liver and uterine tissue was crushed into a fine powder under liquid nitrogen, using a mortar and pestle. A total of 300 mg of crushed rat liver or uterine horn was homogenised in 2 mls of ice-cold 0.9% saline in 30-second bursts. The homogenate was then vortexed in 3 ml 99.9% Propan-2-ol, and left to incubate at room temperature for 5 minutes. After the 5 min incubation period, 6 ml of 98% chloroform was added to the solution, inverted, and centrifuged at 3000 rpm for 10 minutes. Following centrifugation, the chloroform layer was transferred into a fresh tube using a disposable glass pipette and dried under nitrogen gas at 37 °C. Once evaporated, samples were then reconstituted in either 500 μl (liver) or 100 μl (uterine horn) 9:1 Chloroform: Methanol, and 20 μl of this solution was then pipetted onto PUFA COAT^TM^ collection cards^47^ for fatty acid analysis.

### Lipid Analysis

All samples were analysed for their fatty acid profile using a gas chromatography flame ionisation detector (GC-FID) as described previously by Liu *et al*.^47^.

### Statistical Analysis

All the data were analysed using the Statistical Package for Social Science (Version 21; SPSS Inc, Chicago, IL, USA). The homogeneity of the data was assessed and if not normally distributed appropriately transformed to achieve normal distribution. To determine the effect of maternal obesity and change of diet at conception on the plasma, hepatic and uterine fatty acid composition during labour the statistical test used was one-way ANOVA, with LSD post-hoc tests to determine difference between individual treatment groups where the ANOVA revealed significant differences between groups. All data are expressed as the mean value ± SEM and P = ≤0.05 was considered statistically significant.
