# Author Correction: Maternal obesity-induced decreases in plasma, hepatic and uterine polyunsaturated fatty acids during labour is reversed through improved nutrition at conception

**DOI:** 10.1038/s41598-018-33001-0

**Published:** 2018-09-27

**Authors:** Ronan Muir, Ge Liu, Raheela Khan, Anatoly Shmygol, Siobhan Quenby, Robert Alan Gibson, Beverly Muhlhausler, Matthew Elmes

**Affiliations:** 1 Division of Nutritional Science, School of Bioscience, University of Nottingham, Sutton Bonington Campus, Loughborough, LE12 5RD England United Kingdom; 2 Graduate School of Medicine, University of Nottingham, Royal Derby Hospital, Uttoxeter Road, Derby, DE22 3DT England United Kingdom; 3 Department of Physiology, College of Medicine and Health Sciences, United Arab Emirates University, Al Ain, P. O. Box 17666 UAE; 4 Biomedical Research Unit in Reproductive Health, University Hospitals Coventry and Warwickshire NHS Trust, Coventry, CV2 2DX Warwickshire United Kingdom; 5 Healthy Mothers, Babies and Children’s Theme, South Australian Health and Medical Research Institute (SAHMRI), Adelaide, Australia; 6 Department of Wine and Food Science, FOODplus Research Centre, School of Agriculture, Food and Wine, University of Adelaide, Adelaide, Australia

Correction to: *Scientific Reports* 10.1038/s41598-018-21809-9, published online 21 February 2018

The Acknowledgements section in this Article is incorrect.

“We would like to acknowledge the expert technical assistance of Mr R Plant and Mr Mick Baker. We have no conflict of interest. This work was funded by the University of Nottingham BBSRC-DTP PhD studentship, University Hospitals Coventry & Warwickshire NHS Trust studentship [grant number BB/J014508/1] and Rosetrees Trust [grant number M449] and the United Arab Emirates University [Research Grant number 31M311].”

should read:

“This work was supported by the Biotechnology and Biological Sciences Research Council [grant number BB/J014508/1]; University Hospitals Coventry & Warwickshire NHS Trust studentship, Rosetrees Trust [grant number M449] and the United Arab Emirates University [Research Grant number 31M311]. We would also like to acknowledge the expert technical assistance of Mr Richard Plant and Mr Mick Baker”.

